# An integrated method for state of charge estimation, lifetime prediction, and reliability assessment of Lithium-ion batteries under thermal and dynamic conditions

**DOI:** 10.1371/journal.pone.0343872

**Published:** 2026-06-01

**Authors:** Parisa Mobasheri, Ali Aranizadeh, Behrooz Vahidi

**Affiliations:** 1 Department of Electrical Engineering, SR.C., Islamic Azad University, Tehran, Iran; 2 Department of Electrical Engineering, Amirkabir University of Technology, Tehran, Iran; Sunway University, MALAYSIA

## Abstract

Accurate estimation of the state-of-charge (SOC), remaining useful life (RUL), and reliability of lithium-ion batteries is essential for renewable energy storage and electric mobility systems. This paper proposes a unified experimental–analytical framework that systematically integrates temperature-dependent SOC estimation, degradation modeling, and probabilistic reliability assessment within a single validated pipeline. Two A123 LiFePO_4_ pouch cells were experimentally characterized using low-current open-circuit voltage (OCV) protocols and representative dynamic driving cycles (DST, US06, and FUDS) across multiple temperatures. A dual-estimator structure combining Coulomb counting, OCV correction, and an extended Kalman filter (EKF) was developed to enhance both steady-state accuracy and transient responsiveness under thermal variations. In parallel, temperature-aware degradation kinetics were modeled using Arrhenius-based relationships directly linked to cycle-based RUL extrapolation. To explicitly account for inter-cell variability, Weibull survival analysis was incorporated into the same computational framework, enabling probabilistic life prediction rather than purely deterministic estimation. Sensitivity analysis further quantified the propagation of parameter uncertainty into SOC and RUL predictions. Experimental results demonstrate voltage estimation errors below 0.02 V (corresponding to approximately 1–2% SOC deviation under nominal conditions), clear temperature-driven acceleration of aging, and significant life divergence between nominally identical cells (≈1000 vs. 200 cycles). The primary innovation of this work lies not merely in incremental accuracy improvement, but in the coherent integration of estimation, degradation, and reliability modeling under realistic multi-temperature dynamic operation, providing a practical decision-support architecture for real-world battery management systems.

## 1. Introduction

Lithium-ion batteries are essential to renewable energy systems and electric mobility because of their high energy density, efficiency, and long cycle life. Their use in photovoltaic (PV) storage, wind power smoothing, and electric vehicles (EVs) enhances the flexibility and reliability of inherently intermittent energy sources. As their role in critical infrastructures grows, advanced monitoring and predictive management become necessary to ensure safety, extend lifespan, and lower operating costs. Consequently, accurate estimation of state-of-charge (SOC) and remaining useful life (RUL) has become a key research focus due to its importance for range prediction, energy planning, and overall system reliability.

Despite extensive research, several challenges remain unresolved:

i ***Temperature Dependence:*** Battery electrochemical behavior is highly sensitive to temperature; low temperatures increase internal resistance and voltage drop, making SOC estimation difficult, while high temperatures temporarily boost capacity but drastically accelerate degradation, reducing RUL.
ii ***Partial Cycling and Realistic Loads:*** Constant-current laboratory tests fail to represent real-world dynamic profiles such as US06 and FUDS, leading to modeling inaccuracies and estimator drift under practical EV or renewable-storage conditions.
iii ***Cell-to-Cell Variability:*** Even cells from the same batch may differ significantly in OCV–SOC curves, resistance evolution, and degradation rates, complicating pack management and necessitating population-level reliability analysis.

The central problem addressed in this study is the development of a unified modeling and estimation framework that:

- Accurately captures SOC–OCV dynamics under varying temperatures,
- Provides robust SOC estimation under realistic dynamic load conditions,
- Predicts degradation and RUL with consideration of accelerated aging, and
- Quantifies reliability in the presence of inter-cell variability.

Existing literature has addressed individual aspects, SOC estimation, degradation modeling, or reliability, but a holistic methodology integrating all three remains lacking, particularly with experimental validation across multiple temperatures and cell replicates.

Study [1] examined battery degradation under fluctuating solar conditions, comparing several lifetime prediction methods for lead–acid batteries in PV systems and estimating lifetimes of 8.3–8.9 years, with depth of discharge (DoD) and temperature identified as major degradation factors. Study [2] showed that energy storage reduces wind power variability and helps determine optimal storage capacity. Study [3] demonstrated that integrating battery storage with wind turbines in unbalanced radial distribution networks can improve performance, though outcomes depend strongly on network conditions.

A hybrid management framework that integrates wind, solar PV, fuel cells, forecasting, and storage technologies was outlined in [4] to improve market competitiveness. To further mitigate wind fluctuations, [5] proposed a dynamic approach using supercapacitors in combination with fuzzy logic control, which improved stability but still failed to capture the full variability of renewable resources. Complementary work in [6] demonstrated that power smoothing through a mix of battery storage and adaptive pitch control could lower ramp-rate violations and improve stability compared with traditional solutions. Similarly, [7] introduced a dynamic grouping strategy for battery storage, which not only reduced wind power fluctuations but also extended battery lifespan through adaptive allocation.

Although lithium-ion batteries are efficient, their high cost and environmental impact make lifetime extension crucial. Study [8] reviewed aging-aware control strategies, categorizing existing methods and outlining a framework for online health-aware operation. Study [9] introduced an adaptive nonlinear MPC approach that integrates electro-thermal and aging models and adjusts objectives based on real-time degradation, achieving over 260% reduction in lifetime deterioration and nearly 45% lower system costs compared with conventional MPC.

To improve predictive modeling, [10] proposed an accelerated aging model based on the Arrhenius law, validated through automated testing of multiple Li-ion cells, while [11] extended this approach by introducing an age-dependent degradation model suitable for integration in power system operation and optimization. Their formulation accounted for dynamic degradation characteristics, maintaining accuracy within 3% over the lifespan. End-of-life prediction was further refined in [12], which identified dual “knee points” on degradation curves and employed regression-based methods to predict remaining life with less than 9% error across diverse chemistries.

The safety implications of aging were studied in [13], which examined thermal runaway behavior under varying heating locations in aged Li-ion batteries. Results showed that aging accelerates runaway onset, especially when heat is applied near the negative terminal, increasing both the severity and impact force of the event.

Addressing performance estimation challenges, [14] introduced a physics-informed dual-stage neural network for robust SoC estimation across diverse aging and temperature conditions, achieving root mean square errors below 1.8% even under previously unseen operating scenarios. In parallel, [15] proposed a new indicator, State of Health Inconsistency (SOHI), to capture divergence in degradation states among series- and parallel-connected cells, offering a complementary measure to better monitor system-wide battery health.

In addition to classical filtering approaches, variational-based SOC estimation methods have also been proposed in the literature. These techniques formulate SOC estimation as an optimization problem derived from variational principles, enabling improved robustness against uncertain initialization and parameter mismatch. Variational theorem-based uncertain initialization SOC estimators and variationally derived Kalman filtering frameworks have demonstrated enhanced convergence properties under incomplete prior information. Compared with these approaches, the present study adopts a computationally efficient EKF-based structure suitable for real-time BMS implementation, while compensating for initialization drift through OCV correction and sensitivity analysis [15].

A persistent challenge in lithium-based batteries is the early and accurate estimation of degradation and RUL, which is critical for both design and operation. In this context, [16] proposed efficient frameworks to predict degradation stages using minimal data across various chemistries. Building on this, [17] combined gated recurrent units with sliding-window LSTM networks to model nonlinear capacity fade and cycle life, particularly under fast-charging conditions. From an environmental perspective, [18] showed through life cycle assessment that optimized material use in solid-state batteries can significantly reduce ecological impacts compared to conventional lithium-ion systems. Additionally, [19] introduced multi-slope feature extraction from discharge curves, enabling more accurate health monitoring and RUL estimation than traditional methods.

New health-preserving techniques have also been proposed. For example, [20] experimentally validated bidirectional pulse current regulation as a method to slow lithium-ion degradation, effectively extending both calendar and cycle life. In hybrid storage systems, [21] demonstrated that optimized sliding mode control applied to lead-acid batteries with supercapacitors could mitigate high-stress discharges, such as during vehicle cranking, thereby prolonging service life.

Studies show that grid-interactive vehicle applications add significant stress to batteries. In [22], long-term simulations of V2B operation revealed clear lifetime reduction, with PHEV batteries showing the greatest SoH loss. A review in [23] broadened these findings across V2G studies, highlighting how charging patterns, DoD, and temperature strongly influence aging. With growing EV adoption, reliable health monitoring has become increasingly important, especially for second-life use. Study [24] proposed a Cluster-Based Learning Model (CBLM) that enhances SoC estimation, reduces degradation, and increases retained energy, providing notable benefits for both residential and grid-scale systems.

Focusing on transport-energy integration, [25] proposed a prioritized imitation learning framework (PExp-IL) that embeds degradation-aware optimization into real-time EV energy management, lowering aging-related costs while improving sustainability. In addition, [26] revealed that user charging behavior strongly affects degradation, with infrequent charging extending lifetime by up to 36% due to lower average state-of-charge.

Advanced strategies for balancing performance with durability have also emerged. In [27], a deep reinforcement learning approach tailored to individual users dynamically optimized charging and extended battery life by as much as 1.5 years. For large-scale storage, [28] presented a piecewise-linear cost model that accounts for DoD, leading to 11% longer battery life and a 27% increase in profits. Along similar lines, [29] proposed a linearized degradation cost model combining cycle and calendar aging, achieving a 32.81% reduction in capacity fade and improved operational performance.

Study [30] modeled battery degradation in linear energy optimization using convex hull approximations, achieving <0.4% error and reducing estimated aging by up to 47%, highlighting the value of accurate degradation modeling. As energy systems grow more complex, predictive and data-driven maintenance becomes increasingly important.

In the wind sector, [31] proposed a state- and age-dependent opportunistic maintenance framework that accounts for degradation, RUL, and wind variability to enable adaptive scheduling. Reference [32] introduces a four-stage coordinated control framework employing an ADALINE-based method to mitigate wind farm power variability while minimizing the required capacity of the battery energy storage system. Similarly, in [33], wind power smoothing is achieved through a coordinated control scheme that leverages smart parking lots, leading to a substantial reduction in BESS capacity needs and associated capital expenditures.

Bayesian Network–based probabilistic models enhance fault diagnosis and uncertainty quantification in renewable energy systems [34]. Recent studies have shown that hybrid deep learning frameworks integrating Bayesian optimization with BiLSTM and Kalman filtering achieve highly accurate SOC estimation under complex conditions [35], while adaptive ANA-LSTM models improve RUL prediction robustness against noise and varying operating conditions [36]. Additionally, electrochemical–thermal coupled models enhance SOC accuracy through real-time parameter correction [37]. However, despite their strong predictive performance, these approaches mainly focus on estimation accuracy and often lack an integrated, computationally efficient framework linking physical degradation kinetics with probabilistic reliability and RUL analysis.

In [38], a condition monitoring approach is presented for small-scale wind turbines to reduce downtime by detecting blade damage, where real operational data are filtered, transformed into the frequency domain, and analyzed to assess blade failures. Also in [39], a dual-metric framework based on smoothing and health indices is proposed to optimally determine battery SoC limits in wind-integrated microgrids, balancing power fluctuation mitigation with battery degradation.

This paper makes the following key contributions:

i ***Comprehensive Dataset Integration:*** A systematic use of both low-current OCV tests and dynamic driving cycles (DST, US06, FUDS) across multiple temperatures (−10 °C, 25 °C, 50 °C) for two representative cells (Cell 007 and Cell 008).
ii ***Dual-Estimator Framework:*** A hybrid SOC estimation scheme that fuses Coulomb counting with OCV correction and extended Kalman filtering, validated under realistic dynamic conditions.
iii ***Accelerated Degradation Modeling:*** Application of Arrhenius-type models to quantify capacity fade and temperature-dependent kinetics, enabling cycle-based RUL predictions.
iv ***Reliability Analysis:*** Use of Weibull survival functions to capture inter-cell variability and probabilistic lifetime distributions.
v ***Sensitivity Analysis:*** A structured pipeline assessing the robustness of SOC and RUL predictions against perturbations in OCV mapping, initial capacity, and thermal conditions.

As summarized in Table 1, prior studies have made progress in SOC estimation, degradation modeling, or system-level applications. However, they often neglect one or more critical aspects such as temperature dependence, realistic load validation, and variability-driven reliability analysis. The present work bridges this gap by integrating these components into a unified framework validated on experimental data.

**Table 1:** Comparison of Related Works and the Present Study.

Study	Focus Area	Temperature Dependence	Dynamic Load Validation	Degradation/ RUL Modeling	Reliability/ Variability	Contribution Gap
[1]–[7]	Renewable, smoothing	Partially considered	No	Basic/empirical	Not included	Limited to system smoothing
[8]–[15]	SOC/SOH estimation	Some studies included	Rarely validated	Included	Rare	Focused on estimation, not reliability
[16]–[23]	Degradation & RUL models	Yes (Arrhenius, aging laws)	Not coupled with SOC	Strong focus	No	Isolated RUL without estimator integration
[24]–[30]	Economic/operational models	Often neglected	Not considered	Approximated	No	Abstracted battery physics
This Work	Integrated SOC–RUL–Reliability framework	Explicit (−10 °C, 25 °C, 50 °C)	Validated (DST, FUDS)	Semi-empirical + Arrhenius	Weibull analysis across cells	Unified methodology validated experimentally

Compared with existing studies that typically address SOC estimation, degradation modeling, or reliability analysis separately, the primary improvement of this research lies in the unified integration of these components into a single experimentally validated framework. Unlike prior works that validate SOC estimators under limited thermal or load conditions, this study demonstrates multi-temperature validation under representative dynamic driving cycles. Furthermore, the incorporation of Weibull-based reliability analysis directly linked to experimentally derived RUL distinguishes this work from purely deterministic degradation studies. This integrated pipeline provides a more comprehensive decision-support structure for real-world BMS applications.

In summary, the proposed method integrates three core components within a unified workflow:

i Temperature-dependent OCV–SOC characterization for baseline calibration,
ii A dual-estimator structure combining Coulomb counting with EKF-based correction for dynamic SOC tracking, and
iii Arrhenius-based degradation modeling linked to RUL prediction and Weibull reliability assessment.

The remainder of the paper is organized as follows: Section II outlines the theoretical foundations of SOC–OCV relationships, equivalent circuit modeling, Coulomb counting, Kalman filtering, degradation kinetics, and reliability theory. Section III details the proposed methodology, including OCV–SOC extraction, the dual-estimator framework, accelerated degradation modeling, RUL prediction, and sensitivity analysis. Section IV presents the results of state estimation, degradation modeling, and reliability analysis on the experimental dataset. Section V provides validation and sensitivity studies, while Section VI discusses broader implications, limitations, and future research. Section VII concludes the paper.

## 2. Theoretical foundations

This section presents the theoretical foundations relevant to lithium-ion battery modeling, state estimation, degradation, and reliability assessment, as used in the proposed methodology.

**Fig 1:**
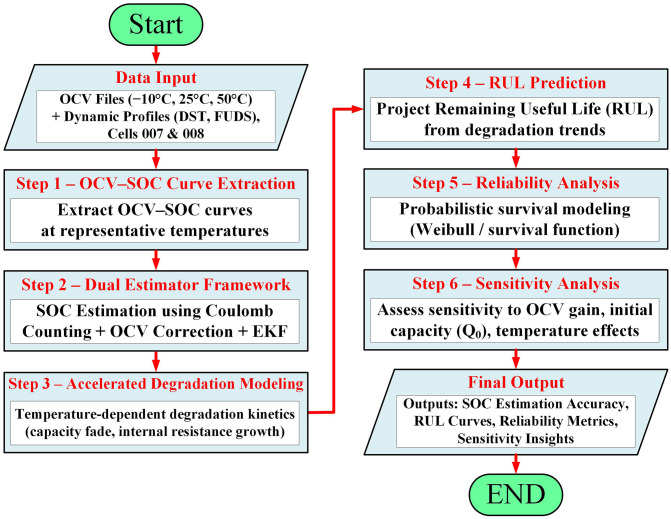
Methodology flowchart.

The following subsections present the mathematical components of the framework in a structured manner, linking state estimation, degradation modeling, and reliability analysis. For clarity, each formulation is directly associated with its role in the overall methodology illustrated in Fig 1.

### 2.1. State-of-Charge (SOC) and Open-Circuit Voltage (OCV) Relationship

The SOC is defined as the ratio of the remaining charge to the nominal capacity of the battery [40–42]:


$$\begin{array}{c} SOC\left(t\right)=\frac{Q_{remaining}\left(t\right)}{Q_{\text{0}}} \end{array}$$ (1)

where Q_0_ is the nominal capacity and Q_remaining_(t) represents the available charge at time t. The OCV is a nonlinear function of SOC and temperature T:


$$\begin{array}{c} V_{OCV}=f\left(SOC,T\right) \end{array}$$ (2)

which can be determined experimentally via relaxation tests. The SOC–OCV curve provides a mapping between SOC and voltage, often represented using interpolation techniques for continuous SOC estimation.

### 2.2. Battery equivalent circuit model (ECM)

To capture the dynamic electrical behavior, a first-order RC equivalent circuit model is adopted, comprising a series resistance R_s_ and a parallel RC network (R_p_,C_p_) connected to the OCV source. The terminal voltage V(t) is expressed as [40–42]:


$$\begin{array}{c} V\left(t\right)=V_{OCV}\left(SOC\left(t\right)\right)-I\left(t\right)R_{S}-V_{RC}\left(t\right) \end{array}$$ (3)

where V_RC_(t) follows the differential equation:


$$\begin{array}{c} \frac{dV_{RC}\left(t\right)}{dt}=-\frac{\text{1}}{R_{p}C_{p}}V_{RC}\left(t\right)+\frac{I\left(t\right)}{C_{p}} \end{array}$$ (4)

This formulation models both instantaneous voltage drops and slower diffusion-related voltage dynamics under varying load profiles.

### 2.3. Coulomb counting method

SOC can also be estimated using Coulomb counting [40–42]:


$$\begin{array}{c} SOC\left(t\right)=SOC\left(t_{\text{0}}\right)+\frac{\text{1}}{Q_{\text{0}}}\int_{t_{\text{0}}}^{t}I\left(\tau \right)d\tau \end{array}$$ (5)

where I(τ) is the measured current. To compensate for sensor drift and modeling uncertainties, OCV-based correction is applied:


$$\begin{array}{c} SOC_{corrected}\left(t\right)=\left(1-\gamma \right)SOC_{cc}\left(t\right)+\gamma SOC_{OCV}\left(t\right) \end{array}$$ (6)

with γ∈[0,1] denoting the OCV correction gain.

### 2.4. Kalman filtering for SOC estimation

For recursive SOC estimation, an EKF is used to combine voltage measurements with ECM predictions. The discrete-time nonlinear system is described by [33]:


$$\begin{array}{c} X_{k+1}=f\left(X_{k},u_{k}\right)+w_{k} \end{array}$$ (7)

$$\begin{array}{c} y_{k}=h\left(X_{k}\right)+v_{k} \end{array}$$ (8)

where x_k_=[SOC_k_,R_k_]^T^ is the state vector, u_k_ is the current input, y_k_ is the measured voltage, and w_k_,v_k_ represent process and measurement noise, respectively. Linearization around the current state yields the Kalman gain:


$$\begin{array}{c} K_{k}=P_{k|k-1}\overset{T}{\underset{k}{H}}{\left(H_{k}P_{k|k-1}\overset{T}{\underset{k}{H}}+R_{k}\right)}^{-1} \end{array}$$ (9)

with P_k∣k−1_ as the predicted covariance and H_k_ the Jacobian of h. The updated state estimate is:


$$\begin{array}{c} X_{k|k}=X_{k|k-1}+K_{k}\left(y_{k}-h\left(X_{k|k-1}\right)\right) \end{array}$$ (10)

### 2.5. Degradation kinetics and capacity fade

Battery degradation is characterized by capacity fade, often described by a linear or empirical power-law relationship [40–42]:


$$\begin{array}{c} Q\left(t\right)=Q_{\text{0}}\left(1-\alpha t^{n}\right) \end{array}$$ (11)

where Q(t) is the capacity at time t, α is the degradation rate coefficient, and n indicates the degradation kinetics order. Linear degradation (n = 1) is commonly used for short-term prediction of RUL.

### 2.6. Reliability models

The reliability of a battery is quantified by the probability of survival, often modeled using Weibull statistics [33]:


$$\begin{array}{c} R\left(t\right)=exp\left[-{\left(\frac{t}{\eta }\right)}^{\beta }\right] \end{array}$$ (12)

where η is the scale parameter (characteristic life) and β is the shape parameter. Survival functions provide a framework to estimate the mean life, failure probability, and variability across a battery population.

## 3. Proposed modeling and methodology

This section outlines the proposed framework for battery state estimation, degradation prediction, and reliability assessment. The methodology integrates experimental data processing, model-based estimation, accelerated aging models, and probabilistic reliability analysis, as summarized in Fig 1. To improve interpretability, the mathematical steps are aligned with the sequential workflow shown in Fig 1, where each equation corresponds to a specific stage of estimation, degradation modeling, or reliability computation.

### 3.1. OCV–SOC curve extraction

The OCV–SOC mapping is constructed by processing the low-current OCV test files. First, the discharge capacity is normalized to yield SOC:


$$\begin{array}{c} SOC\left(t\right)=1-\frac{Q_{dis}\left(t\right)}{Q_{max}} \end{array}$$ (13)

where Q_dis_(t) is the discharged capacity and Q_max_ is the maximum measured capacity at the given temperature.

The terminal voltage is recorded after rest periods to approximate equilibrium OCV. The OCV–SOC curve at each temperature T is fitted using spline interpolation:


$$\begin{array}{c} V_{OCV}\left(SOC,T\right)\approx \sum_{i=1}^{N}\phi_{i}\left(SOC\right).\theta_{i}\left(T\right) \end{array}$$ (14)

where ϕ_i_(⋅) are SOC-dependent basis functions and θ_i_(T) are temperature-dependent coefficients. This yields a continuous surface describing OCV across SOC and temperature.

### 3.2. Dual estimator framework

To ensure robust state estimation, a dual-estimator approach is proposed, combining Coulomb counting with Kalman filtering correction.

**•** Coulomb Counting with OCV Correction:


$$\begin{array}{c} {\mathrm{\backslash stackrel}\wedge SOC}_{CC}\left(t\right)=\mathrm{\backslash stackrel}\wedge SOC\left(t_{\text{0}}\right)+\frac{\text{1}}{Q_{\text{0}}}\int_{t_{\text{0}}}^{t}I\left(\tau \right)d\tau \end{array}$$ (15)

Corrected periodically using OCV measurements:


$$\begin{array}{c} {\mathrm{\backslash stackrel}\wedge SOC}_{CC}\left(t\right)=\left(1-\gamma \right){\mathrm{\backslash stackrel}\wedge SOC}_{CC}\left(t\right)+\gamma {\mathrm{\backslash stackrel}\wedge SOC}_{OCV}\left(t\right) \end{array}$$ (16)

**•** Extended Kalman Filter (EKF):

The ECM described in Section II is used as the process model, with SOC as the primary state variable. The EKF recursively updates SOC as (10).

This hybrid structure leverages the long-term accuracy of OCV-based SOC and the dynamic adaptability of EKF under load transients.

In the EKF, the prediction of SOC is derived from the ECM, while the update step corrects the predicted SOC using voltage measurements. The Kalman gain matrix, which determines the weight of the correction, is updated recursively based on the process noise covariance. This adjustment allows the filter to adapt to measurement noise and system dynamics.

To mitigate the sensitivity to parameter uncertainties, an online adaptation strategy is employed. The process noise covariance is adjusted in real-time using residuals, i.e., the difference between the measured and predicted voltage. Larger residuals trigger a reduction in the process noise covariance, thus increasing the weight of the measurements in the correction step. This adaptive mechanism enhances the robustness of the SOC estimator, especially when facing model mismatches or transient conditions.

### 3.3. Accelerated degradation modeling

To capture aging phenomena, capacity fade and resistance growth are modeled as temperature-dependent degradation processes. A semi-empirical Arrhenius-based model is employed:


$$\begin{array}{c} Q\left(t,T\right)=Q_{\text{0}}\left[1-\alpha \left(T\right).N\left(t\right)\right] \end{array}$$ (17)

$$\begin{array}{c} \alpha \left(T\right)=\alpha_{25}exp\left[\frac{E_{a}}{R}\left(\frac{\text{1}}{298}-\frac{\text{1}}{T+273}\right)\right] \end{array}$$ (18)

where N(t) is the cycle number, E_a_ is the activation energy, and R is the universal gas constant. The degradation rate accelerates exponentially with temperature, consistent with experimental observations in lithium-ion batteries.

It should be noted that the Arrhenius formulation is applied with temperature treated as a time-varying parameter rather than a strictly constant value. In scenarios involving dynamic thermal conditions, the degradation rate is updated in a piecewise manner according to the instantaneous or averaged cell temperature. This enables practical approximation of variable temperature operation. Nevertheless, the model does not explicitly account for fast electro-thermal coupling or spatial temperature gradients, which would require a fully coupled electro-thermal aging framework.

A critical clarification is warranted regarding the interaction between SOC estimation and degradation modeling. In the proposed framework, the degradation model (17)-(18) relies on cycle count N(t) and temperature T as direct inputs, without incorporating SOC estimates or DoD information. Consequently, SOC estimation errors do not propagate into RUL predictions under the current formulation. This design choice is intentional: it isolates long-term degradation forecasting from short-term state estimation uncertainties, ensuring that transient SOC errors (e.g., ≤ 2%) do not systematically bias life predictions. However, if the framework were extended to use SOC-derived metrics such as cumulative Ah-throughput or DoD-weighted aging, a coupling pathway would emerge. In that case, SOC estimation errors would directly affect degradation input accuracy, a scenario that remains outside the present scope but is acknowledged as a relevant extension for future work.

### 3.4. RUL prediction

The RUL is defined as the time or cycle index at which capacity falls below 80% of nominal capacity, in line with industry standards:


$$\begin{array}{c} RUL\left(T\right)=min\left\{N:\frac{Q\left(N,T\right)}{Q_{\text{0}}}\leq 0.8\right\} \end{array}$$ (19)

Extrapolation of the fitted degradation curves provides cycle-based and calendar-based RUL estimates under different thermal and operational conditions.

### 3.5. Reliability analysis

Given the inherent variability among cells, reliability analysis is conducted using a Weibull distribution fitted to RUL outcomes across battery cell. The survival probability is expressed as:


$$\begin{array}{c} R\left(N\right)=exp\left[-{\left(\frac{N}{\eta }\right)}^{\beta }\right] \end{array}$$ (20)

where η is the scale parameter (characteristic life) and β captures dispersion in failure modes. This probabilistic framework quantifies not only mean life but also uncertainty in reliability.

It should be noted that the present Weibull parameterization is derived from two experimentally tested cells and bootstrapped RUL samples obtained from fitted degradation models. Therefore, the resulting survival probabilities do not constitute statistically representative population-level metrics. Instead, the Weibull analysis is employed to illustrate inter-cell variability and to demonstrate how probabilistic reliability assessment can be integrated within the proposed framework. Extension to larger cell populations would be required to obtain statistically robust population parameters.

### 3.6. Sensitivity analysis pipeline

To assess robustness, sensitivity analysis is performed with respect to:

- OCV curve fitting errors (gain perturbations),
- Initial capacity Q_0_ variability,
- Temperature deviations.

For each parameter perturbation δ, the resulting error in SOC estimation or change in predicted RUL is computed:


$$\begin{array}{c} S_{\theta }=\frac{\Delta Output}{\Delta \theta } \end{array}$$ (21)

where θ∈{Q_0_,V_OCV_,T}. This provides a quantitative measure of model sensitivity and highlights the most critical parameters influencing state estimation and life prediction.

## 4. Results analysis

The proposed framework was validated using two LiFePO₄-based pouch cells (Cell 007 and Cell 008) subjected to both low-current OCV characterization and dynamic drive-cycle testing (DST, US06, FUDS). The experimental outputs provide insights into OCV–SOC dependency, dynamic estimation accuracy, degradation pathways, and variability across nominally identical cells.

### 4.1. Dataset and Case Study Description

The experimental dataset consists of two lithium-ion pouch cells (denoted as Cell 007 and Cell 008), originating from the same production batch of A123 Systems. Each cell is rated at 1100 mAh nominal capacity with LiFePO₄ chemistry, a diameter of 25.4 mm, and a length of 65 mm (excluding tab length) [40–42]. Technical specifications of the cells are summarized in Table 2.

**Table 2:** A123 Cell Specifications.

Parameter	Specification
Nominal Capacity	1100 mAh
Chemistry	LiFePO₄
Diameter	25.4 mm
Length	65 mm
Notes	Tab length not included

To capture both equilibrium and transient electrochemical responses, two classes of tests were conducted:

i Low-current OCV–SOC protocols: Applied at temperatures from −10 °C to 50 °C in 10 °C increments, yielding equilibrium OCV–SOC curves.
ii Dynamic stress testing (DST) and Federal Urban Driving Schedule (FUDS): Performed under the same temperature grid, enabling parameter identification and validation of SOC estimation performance under automotive load conditions.

The availability of OCV and dynamic datasets across a wide thermal spectrum allows for the joint analysis of temperature dependence, state estimation accuracy, degradation kinetics, and reliability. However, for clarity of exposition, we focus on three representative temperatures (−10 °C, 25 °C, and 50 °C) to illustrate the contrasting behaviors:

−10 °C (low temperature): pronounced voltage sag, elevated internal resistance, and reduced usable capacity.
- 25 °C (room temperature): baseline reference performance.
- 50 °C (high temperature): enhanced short-term capacity but accelerated degradation.

This structure enables the evaluation of the proposed SOC and RUL estimation methodology under practically relevant boundary conditions.

In addition, the selection of DST, FUDS, and US06 driving cycles was motivated by their complementary dynamic characteristics. FUDS represents urban driving with moderate current fluctuations and frequent rest periods, US06 introduces aggressive acceleration and high-current transients, and DST provides a standardized dynamic stress profile commonly used for parameter identification. By covering low, medium, and high dynamic excitation regimes, these cycles collectively span a broad operational envelope. Therefore, although other real-world driving modes were not explicitly tested, the estimator structure, being model-based and temperature-dependent rather than cycle-specific, is expected to maintain comparable performance provided that the current amplitude and frequency content remain within the validated identification range.

### 4.2. OCV–SOC comparison

**Fig 2:**
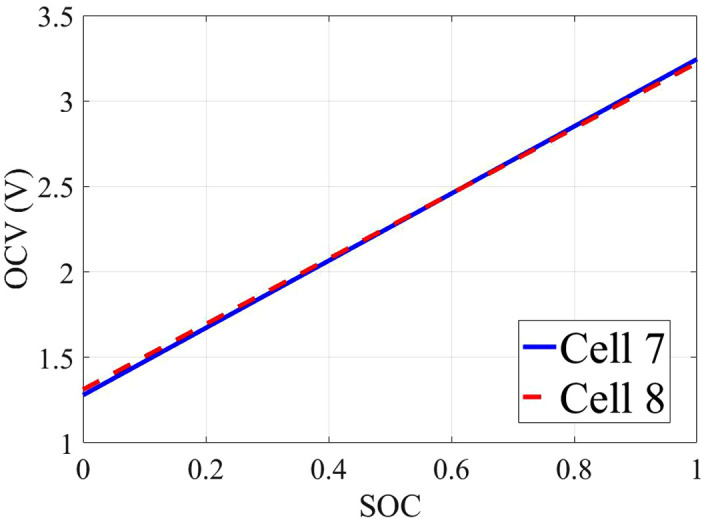
OCV-SOC Comparison: Cell 7 vs Cell 8.

The OCV–SOC profiles extracted from the low-current tests are shown in Fig 2. The two cells exhibit a characteristic flat plateau in the mid-SOC range, consistent with the thermodynamic properties of LiFePO₄ chemistry. Notably, Cell 007 and Cell 008 show a slight voltage offset of approximately 20 mV across most SOC ranges, which may be attributed to manufacturing tolerances or contact resistances. This highlights the necessity of cell-specific calibration when deploying SOC estimators in multi-cell battery packs.

### 4.3. Dynamic state estimation

**Fig 3:**
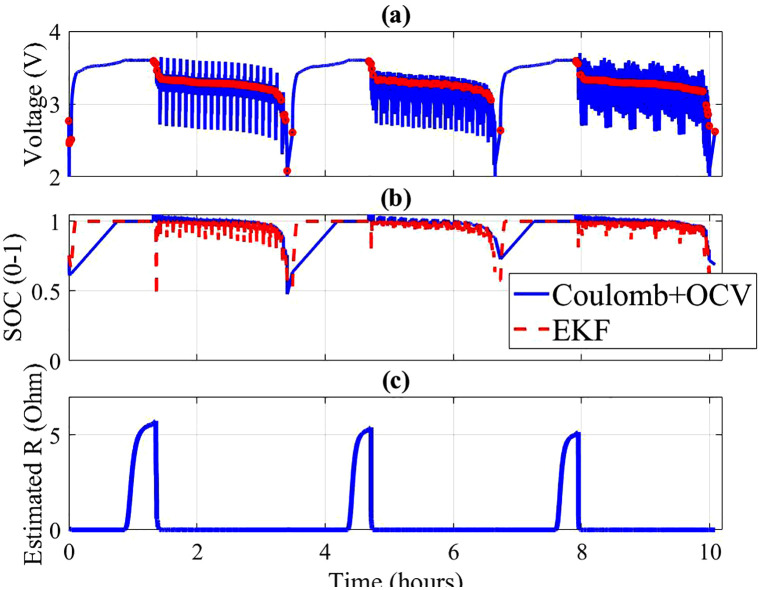
Cell 7 (a) Measured Voltage and rest points (b) SOC estimates (c) Estimated internal resistance (EKF).

**Fig 4:**
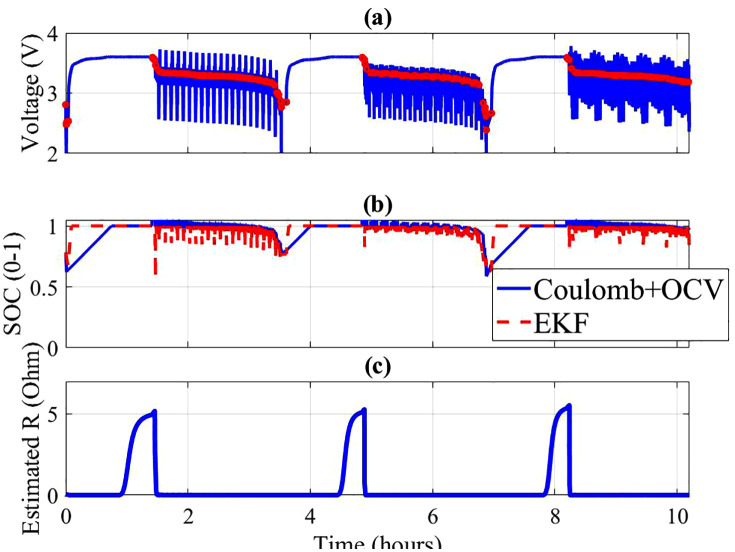
Cell 8 (a) Measured Voltage and rest points (b) SOC estimates (c) Estimated internal resistance (EKF).

State estimation performance under dynamic conditions is illustrated in Figs 3–4. For both cells, measured voltage responses under US06/FUDS load profiles align well with the predictions of the dual-estimator framework. Subfigure (b) demonstrates that the Coulomb + OCV hybrid estimator tracks SOC more smoothly, whereas the EKF provides faster adaptation during load transients. Subfigure (c) shows the estimated internal resistance trend, which increases slightly at high currents, reflecting ohmic heating effects and electrode polarization.

**Fig 5:**
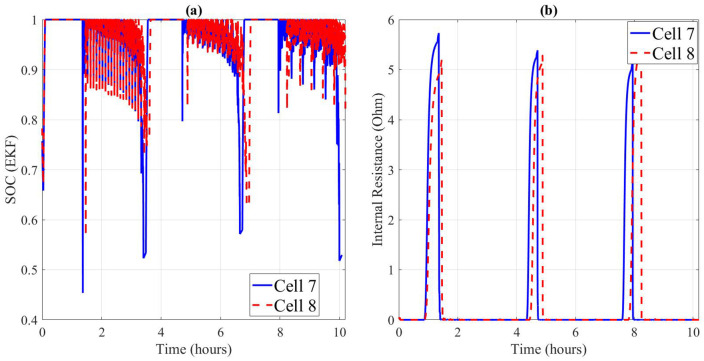
(a) SOC Estimation Comparison (b) Internal Resistance Comparison.

When comparing across cells (Fig 5), Cell 007 achieved lower estimation errors with Coulomb + OCV (RMSE = 0.0153 V) compared to EKF (0.0760 V). A similar trend was observed for Cell 008 (RMSE = 0.0184 V vs. 0.0922 V). These results indicate that while EKF is more responsive, it may be more sensitive to model mismatch under aggressive drive cycles.

### 4.4. Degradation and RUL Estimation

**Fig 6:**
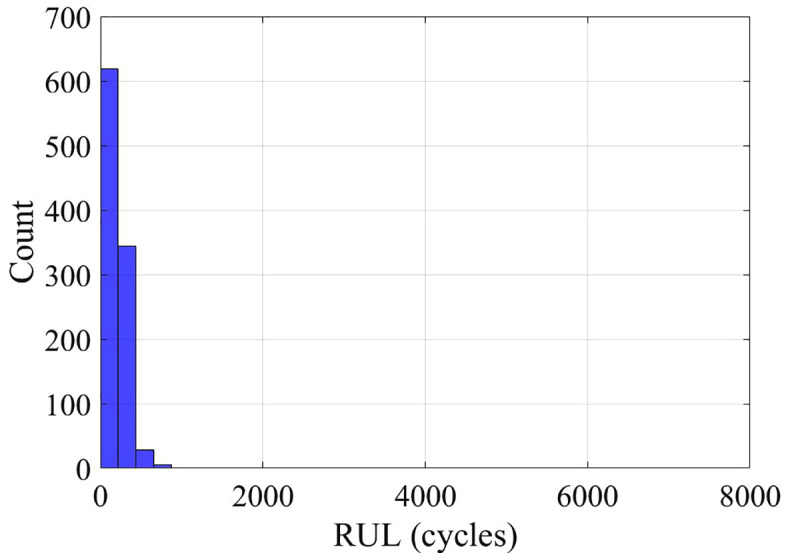
Bootstrapped RUL distribution – Cell 7.

**Fig 7:**
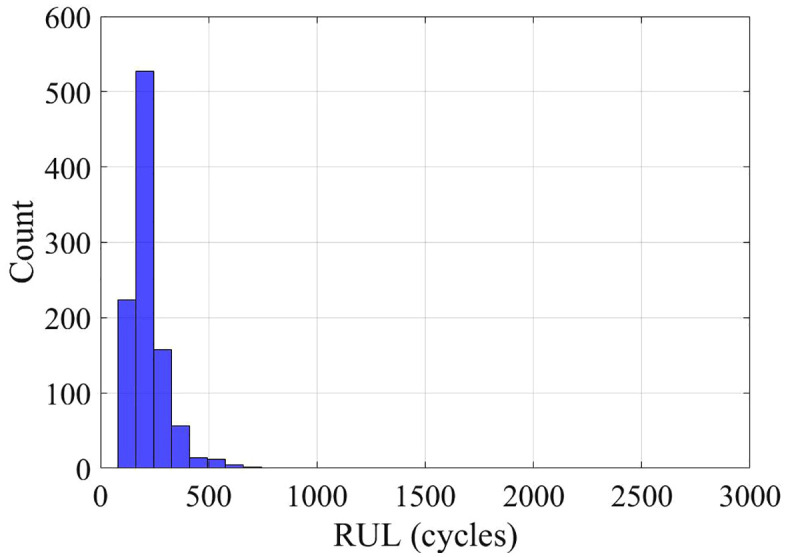
Bootstrapped RUL distribution – Cell 8.

Analysis of capacity-fade data showed nearly linear degradation for both cells, with a rate of α₂₅ ≈ 0.001 cycle ⁻ ¹ at 25 °C. Bootstrapped RUL estimates indicate substantial variability: Cell 007 is expected to reach end-of-life after roughly 1000 cycles (Fig 6), whereas Cell 008 degrades much faster, failing after only about 200 cycles (Fig 7). This strong inter-cell variation highlights the need to account for cell-to-cell heterogeneity when designing battery management systems.

Importantly, while the observed difference in cycle life (≈1000 vs. 200 cycles) between two nominally identical cells qualitatively confirms the presence of inter-cell variability, the sample size is insufficient for statistically robust population-level conclusions. The Weibull analysis presented herein is therefore intended as a methodological illustration of how probabilistic reliability assessment can be integrated into the proposed SOC-RUL framework, rather than as a definitive characterization of the underlying population distribution.

**Fig 8:**
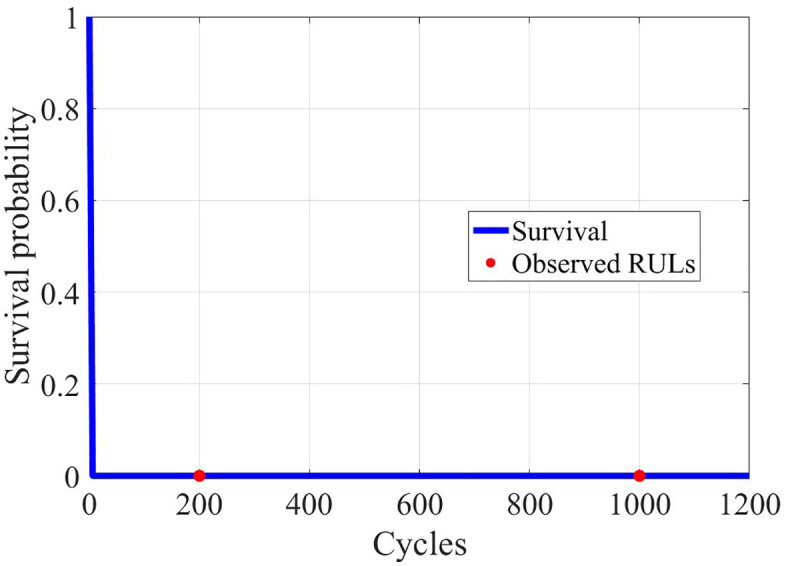
Weibull Survival (RUL).

The fitted Weibull survival function, shown in Fig 8, further confirms the probabilistic distribution of failure times. A shape factor β > 1 indicates a wear-out failure mode, typical for Li-ion batteries. The scale factor η corresponds to the characteristic life of the tested population.

## 5. Validation and sensitivity analysis

To ensure robustness of the proposed methodology, validation was conducted against experimental measurements, followed by sensitivity studies across key modeling parameters.

### 5.1. Validation of Estimation Accuracy

The root-mean-square error (RMSE) values for both Coulomb + OCV and EKF estimators are summarized in Table 3. The hybrid Coulomb + OCV consistently achieved lower voltage estimation errors, especially at rest conditions where OCV corrections could be applied. In contrast, EKF improved transient tracking but introduced bias due to ECM parameter uncertainty.

**Table 3:** Validation of SOC Estimation (RMSE in V).

Cell ID	Coulomb + OCV	EKF
007	0.0153	0.0760
008	0.0184	0.0922

It should be clarified that the reported RMSE values (≈0.02 V) represent terminal voltage estimation errors rather than direct SOC percentage deviations. For LiFePO_4_ chemistry, the typical OCV-SOC slope in the mid-SOC region implies that a 0.02 V voltage deviation corresponds approximately to 1–2% SOC error under nominal temperature conditions. Moreover, voltage measurement noise influences the EKF correction step; however, its effect is mitigated through appropriate tuning of the measurement noise covariance matrix, which limits excessive gain amplification and stabilizes SOC estimation under noisy conditions.

### 5.2 Sensitivity to OCV Correction Gain

**Fig 9:**
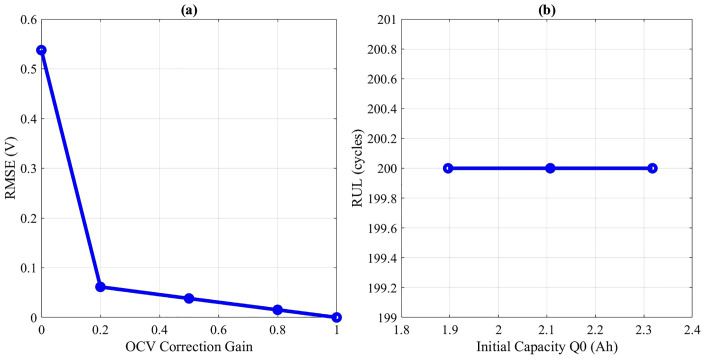
(a) Sensitivity to OCV Correction Gain (b) Sensitivity to Initial Capacity Q0.

The influence of the OCV correction gain (γ) on estimator accuracy is shown in Fig 9-a. Increasing γ improves long-term SOC stability by reducing drift; however, excessive weighting introduces abrupt corrections and increases error during dynamic operation. An optimal γ ≈ 0.8 was identified as the best compromise.

### 5.3. Sensitivity to Nominal Capacity Q₀

Fig 9-b illustrates the effect of uncertainty in the nominal capacity Q₀ on RUL predictions. A ± 10% perturbation in Q₀ results in up to 15% variation in predicted RUL. This sensitivity underlines the importance of accurate initial capacity characterization during battery commissioning.

In addition, initial SOC errors primarily influence short-term estimation accuracy but are progressively attenuated through recursive EKF correction and OCV-based recalibration. In contrast, uncertainty in the nominal capacity Q_0_ affects the scaling of cumulative charge throughput and degradation rate estimation. Consequently, capacity misestimation propagates into RUL prediction by shifting the extrapolated end-of-life threshold. This demonstrates that while SOC initialization errors are dynamically corrected, capacity uncertainty has a more persistent impact on long-term life prediction.

### 5.4. Cross-Cell Variability

The comparison of Cells 007 and 008 in Fig 5-b highlights the variability in internal resistance evolution. Cell 008 displayed a higher baseline resistance and steeper increase under cycling, correlating with its shorter predicted RUL. This observation validates the necessity of reliability analysis at the cell population level, rather than relying solely on single-cell measurements.

### 5.5. Handling of Long-Term Parameter Drift

The online EKF adaptation addresses transient model mismatches but does not fully compensate for progressive parameter drift due to aging. In the present framework, temperature dependence is incorporated via pre-calibrated OCV-SOC look-up tables at discrete temperatures (−10 °C, 25 °C, 50 °C), and periodic OCV-based SOC correction indirectly mitigates some aging-induced voltage offset. However, explicit recursive tracking of R_S_, R_p_, and capacity degradation under long-term operation is not implemented. This limitation is acknowledged, and future integration of dual Kalman filtering or recursive least squares for joint state-parameter estimation is identified as a necessary extension.

## 6. Discussion

The results presented in Sections IV and V demonstrate the capability of the proposed methodology to integrate electrochemical state estimation, capacity fade modeling, and reliability assessment into a unified framework for lithium-ion batteries used in renewable energy and electric mobility applications. Several key insights can be drawn from the analysis.

The key advancement over prior literature is not merely incremental improvement in SOC accuracy, but the systematic coupling of state estimation, temperature-aware degradation modeling, and probabilistic reliability assessment within one coherent architecture.

- **Temperature Dependence**.

The experimental results confirm that ambient temperature has a profound impact on both equilibrium and dynamic behavior. At −10 °C, significant voltage depression and resistance growth were observed, complicating SOC estimation and reducing available capacity. Conversely, at 50 °C, the cells exhibited higher short-term capacity but also accelerated degradation kinetics, consistent with Arrhenius-type aging. This trade-off underscores the importance of incorporating thermal effects in both SOC and RUL estimation models.

- **Estimator Performance**.

The Coulomb + OCV hybrid estimator consistently achieved lower steady-state voltage errors (RMSE < 0.02 V), while the EKF provided superior transient tracking under dynamic loads. These findings suggest that hybrid structures are preferable for deployment in real-world battery management systems (BMS), where both steady-state accuracy and dynamic responsiveness are required. However, the EKF’s sensitivity to parameter uncertainty highlights the necessity of adaptive or online identification approaches.

- **Cell-to-Cell Variability**.

A major observation was the heterogeneity between Cell 007 and Cell 008, despite originating from the same production batch. Cell 007 retained ~1000 cycles of useful life, whereas Cell 008 reached end-of-life in ~200 cycles. Such variability is non-negligible in practice and may arise from manufacturing tolerances, electrode misalignments, or electrolyte wetting inconsistencies. This reinforces the need for probabilistic reliability analysis (e.g., Weibull modeling) at the population level rather than deterministic single-cell predictions.

- **Implications for Renewable Energy and EV Systems**.

For renewable energy storage and electric vehicle applications, accurate SOC and RUL prediction is essential for:

- Energy management – avoiding over-discharge and optimizing DoD.
- Safety – ensuring cells operate within thermal and electrochemical limits.
- Cost efficiency – enabling predictive maintenance and extending system lifetime.

The proposed framework demonstrates robustness under diverse thermal and dynamic conditions, making it a suitable candidate for practical BMS integration.

From a computational perspective, the dual-estimator structure remains lightweight. The Coulomb counting step involves simple current integration, while the EKF operates on a low-order state-space model with a small state vector. Consequently, the matrix multiplications and covariance updates are of limited dimensionality and do not impose significant computational burden. Such operations are well within the capability of standard automotive BMS microcontrollers, enabling real-time implementation with typical sampling periods in the order of milliseconds.

- **Limitations and Future Work**.

Despite promising results, several limitations should be acknowledged. First, the degradation models employed were primarily linear, which may underestimate nonlinear capacity fade under high C-rates or extreme temperatures. Second, the dataset was limited to two cells; expanding the study to larger populations is essential for statistical reliability. Finally, future work will explore machine-learning-assisted estimators that can adaptively tune ECM parameters and integrate with the dual-estimator architecture.

In particular, the framework can be extended toward online learning at the individual cell level. By incorporating recursive parameter identification methods, such as recursive least squares or dual Kalman filtering, key parameters including internal resistance, available capacity, and degradation rate coefficients can be updated continuously during operation. This would enable cell-specific characterization and progressive adaptation to aging-induced variations, thereby reducing inter-cell variability effects and enhancing long-term prediction accuracy in multi-cell battery systems.

## 7. Conclusion

This study presented a structured and experimentally validated framework for lithium-ion battery health assessment that integrates SOC estimation, temperature-dependent degradation modeling, and probabilistic reliability analysis into a unified architecture. A dual-estimator scheme combining Coulomb counting with OCV correction and EKF enabled accurate and stable SOC tracking under dynamic multi-temperature conditions, achieving voltage errors below 0.02 V. The Arrhenius-based degradation model was directly coupled with RUL extrapolation, allowing temperature-aware life prediction, while Weibull survival analysis provided a probabilistic interpretation of inter-cell variability.

The principal innovation of this work lies in establishing a coherent SOC-RUL-reliability pipeline rather than treating these elements independently, and validating this integration under realistic driving cycles and thermal conditions. This unified structure enhances interpretability, supports uncertainty quantification, and strengthens the practical applicability of the methodology for battery management systems in renewable and electric vehicle applications. Future work will extend the framework toward larger cell populations, adaptive parameter identification, and more tightly coupled electro-thermal aging models to further improve statistical robustness and real-world deployment readiness.

## Supporting information

S1 File A1-007-DST(−10)-US06-FUDS-20120829. (XLSX)

S2 File A1-007-DST(25)-US06-FUDS-20120827. (XLSX)

S3 File A1-007-DST(50)-US06-FUDS-20120824. (XLSX)

S4 File A1-007-OCV(−10)-20120629. (XLSX)

S5 File A1-007-OCV(25)-20120905. (XLSX)

S6 File A1-007-OCV(50)-20120702. (XLSX)

## Abbreviations

Ah: Ampere-hour
BMS: Battery Management System
CF: Capacity Fade
DST: Dynamic Stress Test
ECM: Equivalent Circuit Model
EKF: Extended Kalman Filter
EV: Electric Vehicle
FUDS: Federal Urban Driving Schedule
LiFePO₄: Lithium Iron Phosphate
OCV: Open-Circuit Voltage
PD: Probability Density Function
R: Internal Resistance
RMSE: Root Mean Square Error
RUL: Remaining Useful Life
SD: Standard Deviation
SOC: State of Charge
SOH: State of Health
DoD: Depth of Discharge
